# Effect of Chokeberry (*Aronia melanocarpa*) Extracts on the Physicochemical Properties of Wheat Starch Pastes and Gels Stored Under Refrigerated Conditions

**DOI:** 10.3390/molecules30214213

**Published:** 2025-10-28

**Authors:** Greta Adamczyk, Agata Maria Pawłowska, Inna Bobel, Artur Szwengiel, Magdalena Krystyjan

**Affiliations:** 1 Department of Food Technology and Human Nutrition, Institute of Food Technology and Nutrition, University of Rzeszow, Zelwerowicza St. 4, 35-601 Rzeszów, Poland; agpawlowska@ur.edu.pl; 2 Department of Bakery and Confectionery Goods Technologies, Educational and Scientific Institute of Food Technology, National University of Food Technologies, 68 Volodymyrska St., 01601 Kyiv, Ukraine; inna_3@ukr.net; 3 Department of Food Technology of Plant Origin, Faculty of Food Science and Nutrition, Poznań University of Life Sciences, Wojska Polskiego St. 31, 60-624 Poznań, Poland; artur.szwengiel@up.poznan.pl; 4 Department of Carbohydrate Technology and Cereal Processing, Faculty of Food Technology, University of Agriculture in Krakow, Mickiewicza Ave. 21, 31-120 Kraków, Poland; magdalena.krystyjan@urk.edu.pl

**Keywords:** wheat starch, black chokeberry, gels, polyphenolic compounds, antioxidant properties, texture

## Abstract

Wheat starch is among the most widely used ingredients in food products. Adding phytochemicals to wheat starch-based foods impacts their properties during processing and influences their quality during storage. This research aimed to investigate the impact of aqueous extract from chokeberry fruits on pasting and textural properties of starch pastes/gels. The extracts from chokeberry with different total extract content (0 and 7 °Brix) were obtained and applied at various doses (10, 20, and 30% *w*/*w*) as a natural additive to the 5% (*w*/*w*) wheat starch suspension. Furthermore, the obtained starch gels with chokeberry extracts were stored for 14 days at 4 °C. The pasting characteristic process showed that wheat starch pastes containing chokeberry extracts (0 and 7 °Brix) had a higher tendency towards retrogradation. Moreover, the results of the texture analysis confirmed this observation because the hardness values of the wheat starch gels with chokeberry extracts were higher compared to starch gels without the extract (during their 14-day storage). On the other hand, the stability of the gels during storage was also determined by the form of the extract used. The course of changes in hardness values observed during storage indicated that the sugar contained in the extract contributed to smaller fluctuations in these changes. Such observations are important from the point of view of designing starch-based gels that are subjected to storage under refrigerated conditions.

## 1. Introduction

*Aronia melanocarpa* (Michx.) Elliott (black chokeberry, Aronia noir) is widely used in food as a natural source of dyes and bioactive compounds. Chokeberries contain antioxidant compounds, including anthocyanins, flavonols, phenolic acids, and tannins, as well as vitamins (C, B2, B6, E, P, PP), sugars, and minerals (Mo, Mn, Cu, B, I, Co). Anthocyanins, which determine their intense dark color, and other polyphenolic compounds contained in chokeberries have strong antioxidant properties. Among the polyphenols present in chokeberries, compounds belonging to three groups can be distinguished: procyanidins, anthocyanins, and phenolic [1,2,3]. Besides bioactive compounds, chokeberry contains significant amounts of sugars. Variable extraction conditions and methods enable the production of extracts with varying total soluble solids content, expressed in °Brix. Variations in extract content can affect the rheological and textural properties, color, and stability of systems during storage. Therefore, it is essential to consider not only the polyphenol content but also the sugars in studies examining the impact of chokeberry extracts on food products [2].

Polyphenols in chokeberries are compounds that exhibit many beneficial health properties. Consuming chokeberry fruits in various forms (fresh, dried, frozen, or as fruit preserves) has proven beneficial effects on the human body, including a regulatory impact on the circulatory system, lipid-lowering, gastroprotective, hepatoprotective, antiviral, anti-aging, anti-inflammatory, and anti-carcinogenic activities, and a potential role in the control of type 2 diabetes [4]. Moreover, phenolic extracts can enhance the shelf-life of food systems through their antioxidant or antimicrobial properties [5].

Starch, as an important ingredient in many food products, influences both quality and storage stability of food. Wheat starch is very popular and consumed as a food ingredient. Common wheat starch contains 18.2–28.8% amylose [6], and its properties are affected by many factors, including the molecular structure of amylose and amylopectin and the presence of accompanying components [7,8]. The susceptibility of native starches to retrogradation has made their modifiers better known as additives that determine the texture of food products. However, consumers’ negative attitudes toward modified substances in food mean that native starches and other natural polysaccharide hydrocolloids (xanthan gum, guar gum, etc.) still receive a lot of attention from food manufacturers. Currently, the possibility of replacing chemically modified starches with natural systems is being sought, and a typical example is polysaccharide hydrocolloids (xanthan gum, guar gum, etc.) [7,9]. But in recent years, much attention has been paid to the effects of phenolic compounds on the properties of starch-based systems [10,11,12,13]. Literature data confirm the impact of phenolic compounds contained in plant extracts on the properties and structure of starch-based food products. Particular attention has been paid to the effects of phenolic compounds extracted from tea and apples on the starch pastes and gels’ properties [14,15,16,17,18,19,20,21,22,23,24,25,26]. Zhu et al. (2008) [27] investigated pasting and textural properties of wheat starch in the presence of 25 phytochemicals (including phenolic acids, flavonoids, coumarins, stilbenes, and tannins). But there are few studies that have observed the interaction of wheat starch gels with complex phytochemical systems where the solution is a mix of substances; e.g., Zhu et al. (2009) [28] studied interactions of phytochemical extracts (pomegranate peel, green tea, Chinese gall, and Chinese hawthorn) with wheat starch.

Modifying starch by interacting with bioactive compounds, including anthocyanins, can help develop food for specific purposes. Additionally, such modifications may affect the stability of starch-based products during storage. No reports have been found in the literature regarding interactions between wheat starch gels and a system consisting of a mixture of compounds found in chokeberry extract, despite their potential technological and functional importance. In our study, aqueous extracts from chokeberry fruits with different total extract contents (0 and 7 °Brix) were obtained and applied as a natural additive to the 5% (*w*/*w*) wheat starch suspension. Moreover, the effect of different doses (10, 20, and 30% *w*/*w*) of extracts on the physicochemical properties of starch pastes and gels was investigated. This research provides valuable insights into the texture of starch-based food products containing bioactive compounds from chokeberry, while also considering the influence of complex systems, such as sugar–polyphenolic compound systems.

## 2. Results and Discussion

### 2.1. LC-MS Analysis of Chokeberry Extract Compounds

Chokeberry fruits are nutritionally dense and rich in bioactive compounds, particularly polyphenols, which contribute to their nutritional and health benefits. This chemical composition makes them a valuable addition to the diet, for their potential to support overall health and prevent chronic diseases [29,30].

The chromatographic analyses of the alcoholic extract of the black chokeberry fruits (*Aronia melanocarpa*) confirmed the presence of several compound classes (Table 1 and Table S1), of which flavonoid glycosides (including anthocyanins) were found to be the predominant and the most diversified group. Alcohols and polyols were the second most abundant group. This is in accordance with the previous findings [4,31,32].

### 2.2. Pasting Parameters of Wheat Starch

Wheat starch (WS) was heated in a system containing chokeberry extract (ChE) in two variants, with 0 and 7 °Brix (Table 2), at doses of 10, 20, and 30% (*w*/*w*) of the total sample weight. During the heating and cooling process, the presence of ChE extract in the wheat starch suspension affected its pasting properties.

The addition of chokeberry extract to starch suspension caused a decrease in the temperature at the beginning of starch gelatinization (T_0_). Wheat starch (control sample) started pasting at 93.9 °C. At the same time, in the WS-ChE systems, the initial increase in viscosity occurred below the WS pasting temperature. It was in the ranges of 88.0–89.4 °C (extract containing 0 °Brix) and 84.8–88.0 °C (for extract containing 7 °Brix). Therefore, it can be concluded that the WS-ChE system became viscous at a lower temperature than in the case of the starch sample without the addition of extract. This temperature was lower at a higher share of ChE in the starch suspension (in the 5%WS30%ChE0°Brix—88.0 °C and in the 5%WS30%ChE7°Brix—84.8 °C). It should also be noted that the sugars in the extract (samples with 7 °Brix) also influenced the T_0_ parameter; hence, these samples were characterized by lower T_0_ values than samples with a content of 0 °Brix. 

The obtained wheat starch paste (5%WS) was characterized by the lowest viscosity, as indicated by the η_max_ (peak viscosity). This viscosity was 22.5 BU in the control sample, while in the samples with ChE, the values varied from 34.5 to 41.5 BU (in samples with 0 °Brix content) and from 45.5 to 59.5 BU (at 7 °Brix content). The presence of sugar compounds (samples with an extract value of 7 °Brix) led to more viscous systems. According to Woodbury et al. (2022) [33], an increase in peak viscosity was observed because the granule rigidity during heating and mixing increased due to sucrose in the wheat starch–sucrose solution. The sugar not only competed with starch for water but also increased H bonding within the granule. A similar increase in viscosity was observed in the work of Boonkor et al. (2022) [34], where in the presence of sugar and acid, the peak viscosity of starches (tapioca and rice) increased by about 40–70% compared to the neutral system. Also, Krystyjan et al. (2022) [35] showed that the starch pasting process depends on the availability of water molecules. Hydrocolloids or other components can limit this amount of water due to their high affinity for water. Also, the type of interaction between the components and starch plays an essential role in this process. In one case, we may come to an enhancement of interactions between components, known as a synergistic effect, while in another case, we may have an antagonistic effect. The results indicate a reduction in the temperature of pasting, which is extremely important because less energy is needed to destroy the starch granules. In turn, the significant increase in viscosity suggests that the extract concentrations used are high enough to cause statistically significant changes.

Another factor that should be considered in our system is the presence of bioactive compounds, which also contain acidic compounds, thereby lowering the pH of the solution. The pH of the starch suspensions strongly affects the gelatinization properties of starch [36]. The tested starch suspension (5%WS) had a pH of 7.4. Adding chokeberry extract (with 0 and 7 °Bx) to the starch suspension caused a decrease in pH (5%WS10%ChE had a pH of 4.10, 5%WS20%ChE—pH 3.82, and 5%WS30%ChE—pH 3.79). Therefore, phenolic compounds, including acids, present in the tested solution influenced the course of pasting and gelatinization of wheat starch. When considering the effect of an extract on wheat starch gelatinization, it is important to consider the predominant bioactive compounds in the extract. These are chlorogenic acid and rutin (Table 1). These will determine the nature and direction of changes in starch pastes and gels. In the case of chlorogenic acid, the pH of the solution will be crucial, while rutin only slightly changes the pH (Zhu et al. 2008) [27]. According to Zhu et al. (2009) [28], both chlorogenic acid and rutin increase the maximum viscosity of the starch suspension. However, literature data show that the extent of these changes depends on the type of starch used and the amount of rutin added [37]. Interactions between hydroxyl groups of phenolic acids in water change the water activity and ionic strength of the aqueous solution, which increases and accelerates the solubility of starch. However, this phenomenon occurs at a specific solution pH [13,38]. Moreover, due to their numerous hydroxyl groups, polyphenols can form hydrogen bonds with the hydroxyl side chains of starch molecules [39], which leads to the formation of complexes [40]. This is because during gelatinization, the crystalline region of the starch granules is unpacked and the glucan chains are stretched, resulting in the starch molecules being able to interact with the ligand [10]. However, it should be noted that the structural composition of polyphenols varies within this group due to differences in molecular weight and the number of hydroxyl and methoxyl groups. Consequently, their ability to form complexes with starch can vary [39]. According to many authors, interactions between phenolic compounds and starch during starch gelatinization generally occur in two ways [41,42]. In the first case, a type V inclusion complex is formed. In this arrangement, the phenolic compound is located in the internal hydrophobic helix of amylose, stabilized by hydrophobic interactions [11]. In the second case, the hydroxyl and carbonyl groups of phenolic compounds interact with starch through hydrogen bonds and van der Waals forces, forming intermolecular aggregates [19,43]. Cereal starches, such as wheat starch, can be identified in A-type starch [39], and when combined with polyphenols, the crystal structure of starch changes.

Another parameter, η_50°C_, showed viscosity values of samples after being cooled down to 50 °C. At this temperature, starch paste changes to a gel. More viscous gel samples were created in the systems including 7 °Brix ChE.

The difference between the final viscosity and the minimum viscosity in the thermostating phase (parameter setback, SB) had the lowest value in the control sample (5%WS = 17.0 BU). In samples containing ChE, the SB values were elevated, with the highest values observed in samples with 10 and 20% ChE. According to Funami et al. (2005) [7] and Kumar and Khatkar (2017) [44], the increase in final viscosity (here η_50°C_) and setback parameter (SB) can show short-term retrogradation of starch gels or indicates gelling ability; thus, a low SB suggests low gelling ability and/or a low degree of retrogradation. In our study, a higher gelling ability and a greater degree of retrogradation of wheat starch were observed in the starch samples with chokeberry extracts. Moreover, the high SB value may be the result of the hydrolysis of starch during its gelatinization in an acidic environment, which results in the susceptibility of starch gels to retrogradation during their storage [34].

Zhu et al. (2009) [28] suggested that those parameters (SB and η_50°C_) can be affected by pH suspension or interaction between the phytochemicals and hydrophobic regions of amylose and the side chains of amylopectin, where hydrogen bonding and van der Waals forces might change the short-term retrogradation and reassociation during the cooling phase. In their research, phytochemical extracts reduced the final viscosity values, but the setback (SB) increased or decreased, and it depended on varied concentrations, chemical compositions, structural diversities, and molecular weights of phytochemicals. The difference between the maximum viscosity value and the viscosity value at the final stage of heating shows parameter BD (breakdown). Parameter BD reflects the stability of the starch granules during the heating process, but also is affected by solid content, e.g., granule remnants and other ingredients (e.g., sugar and acid), amount, and molecular characteristics of soluble [34]. In the wheat starch samples with extract 0 °Brix, BD values were in the range of 0.0–1.0 BU, and with extract 7 °Brix, 0.0–7.5 BU, so higher BD values suggest less starch granule integrity in the presence of sugars. Phenolic compounds did not affect this parameter (Table 2).

### 2.3. Total Phenolic Content and Antioxidant Activity of Gels

The starch pastes prepared in the viscometer were cooled; afterwards, the content of phenolic compounds and antioxidant capacity (using ABTS^+^ and DPPH radicals) of gel samples were assessed during their storing (Table 3). No phenolic content was detected in starch gels without the addition of extract (5% wheat starch gel), and therefore, they had no antioxidant properties. However, the increasing share of chokeberry extract in the starch suspension (from 10 to 30%), both in samples with 0 and 7 °Brix, caused the gel samples to contain phenolic compounds and showed antioxidant properties measured against ABTS^+^ and DPPH radicals.

The ABTS^+^ and DPPH radical scavenging test method showed that fresh gels (0 days) 5%WS10%ChE, 5%WS20%ChE, and 5%WS30%ChE had higher values of free radical scavenging capacity than those stored for 14 days. This is due to the fact that the content of total phenolics in the stored samples was slightly lower than in the fresh samples. This relationship was observed both in gels with an extract containing 0 °Brix and in those with 7 °Brix. The TPCs in fresh samples (5%WS10%ChE0°Brix, 5%WS20%ChE0°Brix, and 5%WS30%ChE0°Brix) were in the range of 20.25–54.97 mgGAE/100 g gel, with stored samples yielding 19.09–53.74 mgGAE/100 g gel. For 5%WS10%ChE7°Brix, 5%WS20%ChE7°Brix, and 5%WS30%ChE7°Brix, the following values were higher and, respectively, obtained: fresh gels—23.09–70.60 mgGAE/100 g gel and stored gels—18.29–67.76 mgGAE/100 g gel. It should be noted that using only methanol for phenolic extraction may yield different recovery efficiencies in fresh vs. stored gels because storage-induced matrix changes (e.g., starch retrogradation and phenolic–matrix interactions) can reduce phenolic extractability. 

### 2.4. Hardness of Gels

The textural properties of wheat starch gel and its mixture with chokeberry extracts with 0 and 7 °Brix in terms of gel hardness were measured during 14 days of refrigeration at 4 °C (Table 4). The hardness of the wheat starch gel on the day of preparation was 0.155N (fresh gel). The presence of chokeberry extract (0 °Brix) in the fresh gel resulted in a significant reduction in the value of this parameter to the range of 0.033–0.037 N and in the samples with 7 °Brix to 0.077–0.031 N. Therefore, the use of chokeberry extract resulted in weak gels obtained on the day of their preparation (fresh gels). So, we can conclude that the use of chokeberry extract, rich in phenolic acids and flavonoids (Table 1), significantly (α = 0.05) reduced the hardness of wheat starch gel. The texture of wheat starch gel can be influenced by various phenolic compounds [25], but the primary factor influencing hardness is pH. This is due to the phenolic acids present in the obtained chokeberry extracts (ChE) (see Table 1), which reduce the gel’s hardness, including compounds such as chlorogenic acid, *p*-coumaric acid, or vanillic acid. According to Zhu et al. (2008) [27], phenolic acids reduce hardness more than the flavonoids and other types of compounds. Moreover, the textural properties of wheat starch gel are affected by structural features of the phenolic compounds. Still, structural influences of the phenolic compounds on the textural parameters of starch gels are not as obvious as those on the pasting parameters of wheat starch [27,28]. Studies show that within the same type of phenolic compounds, significantly different effects on starch hardness were observed. Zhu et al. (2008) [27] investigated the impact of different phenolic acids on the gel properties of wheat starch. The selected acids (3-hydroxybenzoic acid, 4-hydroxybenzoic acid, 2-hydroxycinnamic acid, and 3-hydroxycinnamic acid) had significantly different effects on gel hardness.

During storage, the hardness of 5%WS changed, and on the first and third day of storage, the value successively increased (from 0.256 to 0.348 N); then on the 7th and 10th day, a slight decrease in hardness was noted (0.333 N, 0.308 N), with another increase on the 14th day of storage (0.371 N). A fresh sample gel with a 10% share of 0 °Brix extract (5%WS10%ChE0°Brix) had a low hardness value (0.034 N) on the day of preparation, and then on the 1st day of storage, a significant increase in this parameter was recorded to the value of 0.601 N, decreasing over the next 7 days (0.439 N, 0.381 N). From the 10th to 14th day of storage, it started to increase again and reached the values of 0.354 N and 0.570 N, respectively. Similar relationships were noted in the other two samples (5%WS 20%ChE0°Brix and 5%WS 30%ChE0°Brix): namely, on the 1st day of storage, there was a significant increase in the hardness of the gels, and then the values decreased and increased again after 14 days of storage.

During the storage period, after the gelatinization process, the starch gel starts to retrograde. This phenomenon increases the hardness of the gel because in the first hours, amylose forms a three-dimensional network, and after that, recrystallization of the side chains of amylopectin occurs. The retrogradation phenomenon can be influenced by various factors (pH, water availability, amylose content, gelatinization temperature, and storage temperature) [45,46,47]. The obtained results showed that the extract from chokeberry, which is a complex system with different compounds, influences the wheat starch gel texture during storage (14 days) at refrigeration temperature (4 °C). The wheat starch gel with 7 °Brix extract had lower hardness values on the 14th day of storage (from 312 to 410 N) than the samples with 0 °Brix ChE (455–570 N). This may suggest that the sugars present in the extract stabilize the gel by reducing the retrogradation process because the hardness of the gels is lower than that recorded in the samples with the 0 °Brix extract.

In the case of gel samples containing the addition of an extract (0 and 7 °Brix), a significant increase in the hardness of the gels was observed. Over the 10 days of measurement, samples containing 0 °Brix chokeberry extract tended to decrease gel texture hardness, followed by a subsequent increase on day 14. Conversely, samples containing extract with sugars showed a decreasing trend in hardness values during storage. Therefore, the behavior of the samples containing chokeberry extract was different from that obtained in the gel without the extract, because the 5%WS gel was characterized by the highest hardness on the 14th day of storage, and not on the 1st day, as was the case in the samples containing the extract. In our study we observed, similarly to Zhu et al. (2008) [27] and Zhu et al. (2009) [28], that the variation in structural features of phenolic compounds in starch solution led to different degrees of interaction with amylose and amylopectin, and that phenolic compounds, through hydrogen bonding and van der Waals forces, could not only affect the re-ordering of amylose a few minutes after pasting, but also may influence the retrogradation process of amylose and amylopectin, thus changing the hardness of starch gels to different degrees and at varying times. Additionally, different phytochemical extracts applied in starch solution might weaken the intermolecular interactions between the amylose by inhibiting the junction zone formation, thus decreasing the hardness of the gel texture [28]. In our studies, pasting characteristics indicated that wheat starch gels containing chokeberry extract had a higher tendency for retrogradation. This is indicated by the higher SB parameters of starch samples with the extract than those without it. The texture analysis results confirm this observation because the hardness values of the gels with chokeberry extract were higher compared to starch gels without the extract during their 14-day storage.

### 2.5. Color Parameters

The results of measuring the color parameters of the tested gels stored at 4 °C are presented in Table 5. The tested starch samples with added chokeberry extract (0 and 7 °Brix) on the day of their preparation, as well as during 14 days of storage, were characterized by low values of the L* parameter, compared to the control sample (57.5). Thus, the L* parameter assumed values in the ranges of 28.9–33.7 and 28.8–35.6 in fresh gels. Moreover, storing the samples under cold conditions contributed to a slight increase in these values, which assumed the range of 32.8–44.2 and 31.8–42.9, but the increase in these values was lower than in the case of starch gel without chokeberry (5%WS) (57.5–69.5). Turbidity can be used to characterize the retrogradation behavior of dilute starch pastes (<2%, *w*/*w* starch) [48]. Observing changes in the L* parameter may indicate progression in turbidity in samples where the starch concentration is higher than 1% [49]. In our study, we measured the intensity of lightness using the CIEL*a*b* system. The tested gel samples, without and with the addition of chokeberry extract, were characterized by a lower L* parameter on the day of preparation than on the following days of refrigerated storage. The most significant increase in gel turbidity occurred within 24 h of storage; hence, a significant increase in the L* value occurred at that time. On the following days of storage, the L* parameter continued to increase in value, but to a lesser extent. According to Wang et al. (2006, 2013) [50,51], the first 24 h of storing the starch gels reflect the rapid increase in turbidity. At this time, interactions between amylose chains that were leached out of the granules during gelatinization result in the formation of networks [52,53].

The obtained values of the a* parameter of the starch gel (control sample) on the individual days of the study had negative values, while the starch systems with chokeberry extract had positive values. The color of the starch gels was mainly influenced by the share of the chokeberry fruit additive used, where positive values indicated the predominance of the red color component. Storage of the gels did not significantly affect the change in the a* parameter value.

The value of the b* color parameter of individual samples of wheat starch gels stored for 14 days was also compared. It was shown that the value of this parameter of each gel type changed slightly during storage, depending on the Brix degree value of the fruit extract. Thus, in the 5%WS10%ChE sample containing 0°Brix and 7°Brix extract, the b* color component parameter had negative values. They increased during storage, and in the 5%WS20%ChE and 5%WS30%ChE samples, negative or positive values decreased or increased during storage.

The addition of chokeberry extract noticeably affected the color characteristics of starch-based gels, which is particularly relevant for the formulation of naturally pigmented food products such as fruit gels, dessert fillings, or coatings. The extract with a higher soluble solids content (7 °Brix) produced a deep red–purple coloration and improved color stability compared to the gels containing the 0 °Brix extract. This effect can enhance the sensory attractiveness of clean-label formulations, where the visual appeal and natural origin of pigments are key attributes valued by consumers. Nevertheless, the gradual increase in lightness observed during storage indicates the limited stability of anthocyanins, likely resulting from degradation and polymerization processes. Therefore, maintaining color intensity over time requires the use of stabilizing techniques, for instance, through encapsulation, pH optimization, or the incorporation of co-pigments such as ascorbic acid.

## 3. Materials and Methods

### 3.1. Materials

The native wheat starch (WS) was purchased from Cargill (Schiphol, The Netherlands). WS had the following parameters: 87.9% d.m.; 22.6% amylose; 0.4% protein; and 0.1% lipids. Freeze-dried chokeberry fruits were purchased from LYOFOOD Sp. z o. o. (Kielce, Poland) with the following composition: 3.0% fat, 7.0% proteins, 50.0% carbohydrates (including simple sugars 43.0%), 27.0% fiber, and 0.05% salt.

### 3.2. Methods

#### 3.2.1. LC-MS Analysis

The samples (freeze-dried chokeberry fruits) intended for chromatographic analysis were prepared as follows: the sample was extracted with 70% methanol, 1 g per 5 mL of the extraction mixture. Extractions were carried out for 30 min in an ultrasonic bath (Sonic-1, 80W, Polsonic Palczyński Sp. J., Warszawa, Poland) at a temperature of 20 °C, and then for 30 min on a roller mixer (ChemLand, Stargard, Poland). The samples were centrifuged at 20,000× *g*/20 min (MPW, Warszawa, Poland) and then subjected to LC-MS analysis (Liquid Chromatography–Mass Spectrometry).

Reversed-phase (C18 column) ultra-high-performance liquid chromatography–electrospray ionization–mass spectrometry (RP-UHPLC-ESI-MS) analysis was performed using a Dionex UltiMate 3000 UHPLC (Thermo Fisher Scientific, Sunnyvale, CA, USA) coupled to a Bruker maXis impact ultra-high-resolution orthogonal quadrupole–time-of-flight accelerator (qTOF) equipped with an ESI source and operated in the positive- and negative-ion mode (Bruker Daltonik, Germany). The RP chromatographic separation was achieved with a Kinetex™ 1.7 µm C18 100 Å, LC column 100 × 2.1 mm (Phenomenex, Torrance, CA, USA) according to Biesaga and Pyrzyńska (2013) [54]. The ESI-MS settings were previously described by Mildner-Szkudlarz et al. (2015) [55]. Molecular ions [M + H]^+^ and [M − H]^−^ for phenolic compounds were extracted from full-scan chromatograms (±0.005 *m*/*z*), and peak areas were integrated with TASQ 2.1 (Bruker Daltonik, Bremen, Germany). The limit of quantification (LOQ, where S/N > 15) was determined for caffeic acid, chlorogenic acid, *p*-coumaric acid, synaptic acid, and quercetin, and it was not lower than 0.01 µg/mL.

An analyte-free matrix was not available. Calibration and quality control (QC) samples were prepared in water as a surrogate matrix. The recovery of standards spiked in samples was above 95–106%. The coefficient of determination (R2) for all calibration curves was higher than 0.99. 

The tandem mass spectrometric data were used for searching molecular structures using CSI (Compound Structure Identification): FingerID (Friedrich Schiller University, Jena, Germany). CSI: FingerID is a web service that combines the analysis of isotope patterns in MS spectra with the analysis of fragmentation patterns in MS/MS spectra and predicts molecular fingerprints [56,57,58]. The CANOPUS algorithm was also included to predict compound classes from the molecular fingerprint predicted by CSI: FingerID without any database search involved [58]. It is beneficial structural information when neither spectral nor structural reference data are available. The CANOPUS shows ClassyFire classes [59], and the results table contains compound class (computed with CANOPUS).

#### 3.2.2. Preparation of Chokeberry Extracts

The water-based chokeberry extracts used for preparing starch gel samples were made from freeze-dried fruit. For this purpose, 100.0 g of freeze-dried powdered berries was suspended in 1000 mL of 70% methanol solution. The mixture was extracted using ultrasounds in an ultrasonic bath (Polsonic, Warsaw, Poland) for 30 min at 21 °C. Then, the samples were centrifuged in a laboratory centrifuge (Eppendorf Centrifuge 5430, Hamburg, Germany) at a 7000 rpm for 10 min. Alcohol was removed from the obtained supernatant using a vacuum evaporator (VWR VP 10 Autovac, Wolfertschwenden, Germany). The resulting amount of concentrated chokeberry extract was divided into two equal portions:

(i) The first one was poured into a measuring cup and filled up to 500 mL with distilled water to obtain the aqueous extract of 7 °Brix.

(ii) The second portion of the concentrated chokeberry extract was purified from the sugars using a column containing adsorber LiChroprep RP-18 (40–63 μm) (MilliporeSigma, Burlington, MA, USA), where the sample was washed several times with water until the extract was below 0.1 °Brix. Next, phenolic compounds were eluted from the LiChroprep column with methanol. The obtained alcoholic extract was transferred again to a vacuum evaporator to remove the alcohol. The resulting concentrated aqueous chokeberry extract was poured into a measuring cup and topped up with distilled water to 500 mL. The total extract content was measured again and confirmed to be 0 °Brix. The sugar content in the obtained chokeberry samples was determined by the refractometric method using a PAL-1 digital refractometer from Atago (Tokyo, Japan).

Thus prepared variants of chokeberry extract (0 and 7 °Brix) at different doses (10, 20, and 30% *w*/*w*) were added to 5% (*w*/*w* d.m.) wheat starch suspensions and used for further studies (pasting characteristic, analysis of total polyphenol content and antioxidant properties, and texture analysis).

Additionally, the pH of the obtained extracts was determined using a pH meter (pH-Burette 24, Crison, Barcelona, Spain).

#### 3.2.3. Pasting Characteristic of Suspensions

The measurements of the pasting characteristics of 5% (*w*/*w* d.m.) wheat starch suspensions and their mixtures with 10, 20, and 30% (*w*/*w*) amounts of aqueous extract obtained from freeze-dried chokeberry fruit were taken using the Viscograph-E Brabender (Brabender GmbH & Co. KG, Duisburg, Germany). The measurements of the pasting characteristics of the WS samples and their mixtures with chokeberry extract (ChE) were run with the following parameters: (a) heating from 30 to 95 °C at 1.5 °C/min; (b) maintaining samples at 95 °C for 5 min; and (c) cooling from 95 °C to 50 °C at 1.5 °C/min. The pasting behavior was measured using the Viscograph for 83 min, run in duplicate. During the pasting characteristic process the following parameters were obtained: T_0_ [°C]—temperature at the beginning of pasting; η_max_ (Brabender units) [BU]—maximum viscosity; T_ηmax_ [°C]—temperature at maximum viscosity; η_95°C_ [BU]—viscosity at 95 °C; η_50°C_ [BU]—viscosity after cooling to 50 °C; breakdown (BD) [BU]; and setback (SB) [BU]. Viscograph Data Correlation software (https://www.anton-paar.com/corp-en/products/details/metabridge/?srsltid=AfmBOoqYXwqtRAPwtamnqhtXEmYC6RNYzBZ-aPLoGUR1gU3CyUrmxz9y, accessed on 21 October 2025) was used to process the data (Anton Paar, Duisburg, Germany) [60].

#### 3.2.4. Analysis of Total Polyphenol Content and Antioxidant Properties of Gels

Firstly, the methanol extracts were prepared for analysis of the total phenolic content and antioxidant properties of chokeberry extract and gels. The extraction process was carried out using an ultrasonic bath (Polsonic, Warsaw, Poland) (30 min at 25 °C). The samples were treated with a 70% methanol solution. The obtained supernatants (methanol extracts) were used for further analysis.

The total phenolic content in the obtained gels was assessed using the Folin–Ciocalteu phenol reagent with the method described by Singleton et al. (1999) [61]. The reaction mixture contained extract (0.1 mL), Folin–Ciocalteu reagent (0.2 mL), distilled water (2.0 mL), and 20.0% (*w*/*v*) sodium carbonate solution (1.0 mL). Using a spectrophotometer (Nicolet Evolution 300, Thermo, Waltham, MA, USA), the absorbance of the samples was measured at 765 nm against distilled water. The total phenolic content (TPC) was expressed as gallic acid equivalents (GAEs) in milligrams per 100 g of samples.

According to Re et al. (1999) [62], the antioxidant activity was carried out using the ABTS+ cation radical. The reaction mixture consisted of adding the sample (0.03 mL) and the ABTS+ radical solution to the water (3.0 mL). The absorbance at 734 nm, against distilled water, was measured after 6 min of reaction.

The scavenging activity was measured according to the elimination of DPPH (1,1-diphenyl-2-picrylhydrazyl) free radicals [63]. The reaction mixture consisted of adding the sample (0.5 mL) and the DPPH radical solution to methanol (2.0 mL). The absorbance at 517 nm, against methanol, was measured after 10 min of reaction. The values of the antioxidant activity (ABTS, DPPH) of the studied samples were expressed in mmol TE/100 g (Trolox Equivalent).

Measurements of TPC, ABTS, and DPPH were performed in triplicate using a spectrophotometer (Nicolet Evolution 300, Thermo, Waltham, MA, USA). These measurements were carried out on the day of sample preparation (fresh gels) and on the 14th day of storage.

#### 3.2.5. Textural Properties of Gels

Texture was characterized using a Texture Analyzer EZ-Test EZ-LX (Shimadzu, Tokyo, Japan) equipped with an aluminum probe (d = 7 mm, h = 40 mm), at a force of 20 N. The hardness parameters of the wheat starch gel and its blends with the addition of chokeberry extract were determined using the penetration test according to Adamczyk et al. (2021) [64]. The freshly obtained gels in Viscograph (30 mL) were transferred to plastic cylinders, and hardness was measured for fresh gels (after 1 h of storage) and after 1, 3, 7, 10, and 14 days of storage at 4 °C. Gel hardness was defined as the maximal force applied (N). The reported results were the average values of three replications.

#### 3.2.6. Color Measurement of Gels

Quantitative color measurements were conducted using an UltraScan VIS spectrophotometer (HunterLab, Reston, VA, USA) according to the CIE L*a*b* model, using diffuse/8° geometry with automatic mirroring on and off. The spectrophotometer recorded the parameters L*, a*, and b*, defined as lightness (L* 100 is white and 0 is black), redness+/greenness− (a*), and yellowness+/blueness− (b*). Measurements were made in triplicate [49].

#### 3.2.7. Statistics

The experimental data were calculated using Statistica v.13.3 (StatSoft, Inc., Tulsa, OK, USA). The analysis of variance was performed using Duncan’s test at a confidence level of α = 0.05.

## 4. Conclusions

Introduction of chokeberry extracts (with 0 and 7 °Brix), which are rich in phenolic compounds, significantly influenced the pasting and textural properties of wheat starch pastes and gels. The pasting characteristics and gel texture of wheat starch treated with extract rich in phenolic compounds were affected by factors including the pH and the group of phenolic compounds.

The pasting characteristics indicated that wheat starch pastes containing chokeberry extracts (0 and 7 °Brix) had a higher tendency for retrogradation. This was suggested by the higher SB parameters of starch samples with the extract compared to those without it. The results of the texture analysis confirmed this observation because the hardness values (which can indicate the process of retrogradation) of the gels with chokeberry extracts were higher compared to starch gels without the extract, during their 14-day storage. However, texture analysis also showed that the starch gels with the sugars contained in the extract (samples with 7 °Brix content) resulted in more stable behavior of gels during their 14-day storing, compared to those with 0 °Brix content. This is due to the fact that these tests showed less dynamic changes described by the hardness parameter. Furthermore, it is worth emphasizing that the use of chokeberry extract in freshly prepared samples (fresh gels) contributed to the formation of so-called weak gels. This is because different phytochemical extracts applied in starch solution may weaken the intermolecular interactions between the amylose by inhibiting junction zone formation, thus decreasing the hardness of the gel texture. Of course, this phenomenon should be confirmed in further rheological studies.

In this type of research, it is important to note that the behavior of starch during the pasting process and during storage of gels may depend on the presence of specific groups of compounds in individual samples. Such samples will exhibit different behavior in the environment of phenolic compounds themselves when compared to the presence of particles such as sugars that are part of the extract. This issue is important when using phenolic compounds in the form of extracts to stabilize starch gels during their storage. Further studies on the interaction between starch and plant extracts may provide a scientific basis for phytochemical application in wheat products and for use in special-purpose foods.

## Figures and Tables

**Table 1:** Tentative classification of polyphenols in chokeberry methanol-water extracts. The tandem mass spectrometry data were used for unsupervised prediction.

No.	ClassyFire (Subclass, Probability > 0.950) *	No. of Compounds	Standard Used for Quantification	Concentration ± SD (mg/100 g)
1.	Alcohols and polyols	5	Chlorogenic acid	336.53 ± 16.35
2.	Benzoic acids and derivatives	3	Vanillic acid	11.54 ± 0.37
3.	Biflavonoids and polyflavonoids	2	Catechin	10.55 ± 0.11
4.	Carbohydrates and carbohydrate conjugates	1	*p*-Coumaric acid	1.02 ± 0.05
5.	Chalcones and dihydrochalcones	3	Catechin	2.77 ± 0.08
6.	Flavans	2	Quercetin	34.39 ± 0.56
7.	Flavones	3	Rhamnetin	3.54 ± 0.00
8.	Flavonoid glycosides (including anthocyanins)	31	Rutin	235.55 ± 78.08
9.	Hydroxycinnamic acids and derivatives	7	*p*-Coumaric acid	11.05 ± 0.74
10.	Hydroxyflavonoids	3	Catechin	0.90 ± 0.02
11.	*O*-methylated flavonoids	2	Catechin	0.10 ± 0.01
			Total	647.92 ± 93.96

* Predictions based on MS/MS spectra molecular fingerprints (the CSI: FingerID web service) were used by the CANOPUS algorithm to predict compound classes without involving any database search.

**Table 2:** Parameters of pasting characteristics of starch–extract systems (0 and 7° Brix).

Sample	T_0_ [°C]	η_max_ [BU]	η_95°C_ [BU]	η_50°C_ [BU]	BD [BU]	SB [BU]
Gels with 0 °Brix extract						
5%WS (control)	93.9 ^a^ ± 0.4	22.5 ^b^ ± 0.7	19.0 ^b^ ± 1.4	39.5 ^c^ ± 0.7	0.0 ^a^ ± 0.0	17.0 ^b^ ± 1.4
5%WS10%ChE	89.4 ^b^ ± 0.5	37.0 ^a^ ± 4.2	35.5 ^a^ ± 2.1	81.5 ^ab^ ± 2.1	0.0 ^a^ ± 0.0	44.5 ^a^ ± 2.1
5%WS20%ChE	89.5 ^b^ ± 0.2	34.5 ^a^ ± 0.7	33.0 ^a^ ± 1.4	78.0 ^b^ ± 1.4	0.0 ^a^ ± 0.0	43.5 ^a^ ± 2.1
5%WS30%ChE	88.0 ^b^ ± 2.4	41.5 ^a^ ± 6.4	40.5 ^a^ ± 7.8	83.0 ^a^ ± 0.0	1.0 ^a^ ± 0.0	42.5 ^a^ ± 6.4
Gels with 7 °Brix extract						
5%WS (control)	93.9 ^a^ ± 0.4	22.5 ^c^ ± 0.7	19.0 ^c^ ± 1.4	39.5 ^b^ ± 0.7	0.0 ^b^ ± 0.0	17.0 ^b^ ± 1.4
5%WS10%ChE	88.0 ^b^ ± 0.2	47.0 ^b^ ± 1.4	46.5 ^b^ ± 0.7	91.0 ^a^ ± 1.4	1.5 ^b^ ± 0.7	45.0 ^a^ ± 2.8
5%WS20%ChE	88.1 ^b^ ± 0.1	45.5 ^b^ ± 0.7	44.5 ^b^ ± 0.7	90.0 ^a^ ± 0.0	1.0 ^b^ ± 0.0	45.5 ^a^ ± 0.7
5%WS30%ChE	84.8 ^c^ ± 1.3	59.5 ^a^ ± 6.4	54.5 ^a^ ± 4.9	86.5 ^a^ ± 3.5	7.5 ^a^ ± 2.1	35.0 ^a^ ± 7.1

Parameters in columns denoted with the same letters do not differ significantly at the confidence level α = 0.05.

**Table 3:** Total phenolic content and antioxidant properties in samples with wheat starch and chokeberry extract.

Sample	ABTS [mmolTE/100 g Gel]		DPPH [mmolTE/100 g Gel]		TPC [mgGAE/100 g Gel]	
	Fresh Gels (0 Day)	14 Days of Storage	Fresh Gels (0 Day)	14 Days of Storage	Fresh Gels (0 Day)	14 Days of Storage
	Gels with 0 °Brix extract					
5%WS (control)	0.000 ^d^ ± 0.000	0.000 ^d^ ± 0.000	0.000 ^d^ ± 0.000	0.000 ^d^ ± 0.000	0.00 ^d^ ± 0.00	0.00 ^d^ ± 0.00
5%WS10%ChE	0.244 ^c^ ± 0.008	0.213 ^c^ ± 0.020	0.257 ^c^ ± 0.040	0.145 ^c^ ± 0.008	20.25 ^c^ ± 0.69	19.09 ^c^ ± 1.07
5%WS20%ChE	0.608 ^b^ ± 0.065	0.387 ^b^ ± 0.001	0.448 ^b^ ± 0.147	0.378 ^b^ ± 0.022	35.88 ^b^ ± 2.22	33.11 ^b^ ± 3.74
5%WS30%ChE	2.152 ^a^ ± 0.836	0.969 ^a^ ± 0.041	0.864 ^a^ ± 0.009	0.569 ^a^ ± 0.045	54.97 ^a^ ± 2.42	53.74 ^a^ ± 2.91
	Gels with 7 °Brix extract					
5%WS (control)	0.000 ^d^ ± 0.000	0.000 ^d^ ± 0.000	0.000 ^d^ ± 0.000	0.000 ^d^ ± 0.000	0.00 ^d^ ± 0.00	0.00 ^d^ ± 0.00
5%WS10%ChE	0.263 ^c^ ± 0.006	0.219 ^c^ ± 0.009	0.255 ^c^ ± 0.006	0.136 ^c^ ± 0.004	23.08 ^c^ ± 0.27	18.29 ^c^ ± 1.04
5%WS20%ChE	0.708 ^b^ ± 0.081	0.371 ^b^ ± 0.008	0.600 ^b^ ± 0.018	0.414 ^b^ ± 0.009	34.02 ^b^ ± 1.05	31.59 ^b^ ± 1.03
5%WS30%ChE	1.638 ^a^ ± 0.324	1.309 ^a^ ± 0.085	0.962 ^a^ ± 0.004	0.421 ^a^ ± 0.047	70.60 ^a^ ± 1.48	67.76 ^a^ ± 4.70

Parameters in columns denoted with the same letters do not differ significantly at the confidence level α = 0.05.

**Table 4:** Changes in the hardness of wheat starch gels with 0 and 7 °Brix chokeberry extract during 14-day storage.

Sample	Hardness [N]					
	Fresh Gels (0 Day)	1 Day of Storage	3 Days of Storage	7 Days of Storage	10 Days of Storage	14 Days of Storage
Gels with 0 °Brix extract						
5%WS (control)	0.155 ^a^ ± 0.013	0.256 ^c^ ± 0.038	0.348 ^bc^ ± 0.024	0.333 ^b^ ± 0.033	0.308 ^bc^ ± 0.050	0.371 ^c^ ± 0.018
5%WS 10% ChE	0.034 ^b^ ± 0.003	0.601 ^a^ ± 0.046	0.439 ^a^ ± 0.021	0.381 ^ab^ ± 0.062	0.354 ^b^ ± 0.064	0.570 ^a^ ± 0.021
5%WS 20% ChE	0.033 ^b^ ± 0.001	0.570 ^a^ ± 0.028	0.298 ^c^ ± 0.069	0.462 ^a^ ± 0.007	0.242 ^c^ ± 0.012	0.546 ^ab^ ± 0.096
5%WS 30% ChE	0.037 ^b^ ± 0.007	0.435 ^b^ ± 0.013	0.401 ^ab^ ± 0.032	0.429 ^ab^ ± 0.087	0.448 ^a^ ± 0.031	0.455 ^bc^ ± 0.003
Gels with 7 °Brix extract						
5%WS (control)	0.155 ^a^ ± 0.013	0.265 ^c^ ± 0.038	0.348 ^c^ ± 0.024	0.333 ^c^ ± 0.033	0.308 ^d^ ± 0.050	0.371 ^ab^ ± 0.018
5%WS 10% ChE	0.077 ^b^ ± 0.011	0.528 ^a^ ± 0.010	0.512 ^a^ ± 0.012	0.469 ^a^ ± 0.010	0.433 ^a^ ± 0.016	0.410 ^a^ ± 0.077
5%WS 20% ChE	0.084 ^b^ ± 0.015	0.533 ^a^ ± 0.012	0.421 ^b^ ± 0.004	0.399 ^b^ ± 0.020	0.421 ^b^ ± 0.019	0.312 ^c^ ± 0.026
5%WS 30% ChE	0.031 ^c^ ± 0.002	0.385 ^b^ ± 0.007	0.316 ^d^ ± 0.006	0.403 ^b^ ± 0.010	0.410 ^c^ ± 0.032	0.336 ^ab^ ± 0.025

Parameters in columns denoted with the same letters do not differ significantly at the confidence level α = 0.05.

**Table 5:** Changes in color parameters of wheat starch gels with 0 and 7 °Brix chokeberry extract during 14-day storage.

Sample	Fresh Gels (0 Day)	1 Day of Storage	3 Days of Storage	7 Days of Storage	10 Days of Storage	14 Days of Storage
Gels with 0 °Brix extract						
L*						
5%WS (control)	57.5 ^a^ ± 0.9	65.7 ^a^ ± 0.0	66.2 ^a^ ± 0.1	67.1 ^a^ ± 0.2	67.7 ^a^ ± 0.5	69.5 ^a^ ± 0.5
5%WS10%ChE	33.7 ^b^ ± 0.0	42.1 ^b^ ± 0.3	42.2 ^b^ ± 0.2	43.4 ^b^ ± 0.2	43.8 ^b^ ± 0.1	44.2 ^b^ ± 0.0
5%WS20%ChE	30.6 ^c^ ± 0.0	37.3 ^c^ ± 0.2	36.9 ^c^ ± 0.1	37.3 ^c^ ± 0.0	38.2 ^c^ ± 0.0	38.8 ^c^ ± 0.1
5%WS30%ChE	28.9 ^d^ ± 0.0	32.4 ^d^ ± 0.2	33.4 ^d^ ± 0.1	33.7 ^d^ ± 0.3	33.7 ^d^ ± 0.1	32.8 ^d^ ± 0.0
a*						
5%WS (control)	−1.1 ^c^ ± 0.2	−2.4 ^d^ ± 0.0	−2.4 ^d^ ± 0.0	−2.4 ^c^ ± 0.0	−2.4 ^d^ ± 0.0	−2.5 ^d^ ± 0.0
5%WS10%ChE	10.9 ^a^ ± 0.0	12.8 ^c^ ± 0.2	12.2 ^c^ ± 0.1	12.3 ^b^ ± 0.0	12.6 ^c^ ± 0.0	12.5 ^c^ ± 0.1
5%WS20%ChE	11.0 ^a^ ± 0.0	15.8 ^a^ ± 0.1	15.1 ^b^ ± 0.1	16.3 ^a^ ± 0.0	16.4 ^b^ ± 0.1	16.8 ^b^ ± 0.1
5%WS30%ChE	9.7 ^b^ ± 0.0	14.2 ^b^ ± 0.0	15.9 ^a^ ± 0.2	16.5 ^a^ ± 0.3	16.7 ^a^ ± 0.0	15.1 ^a^ ± 0.0
b*						
5%WS (control)	−3.5 ^c^ ± 0.7	−6.2 ^d^ ± 0.0	−6.5 ^d^ ± 0.0	−6.3 ^d^ ± 0.1	−6.2 ^d^ ± 0.2	−6.6 ^d^ ± 0.0
5%WS10%ChE	−0.3 ^b^ ± 0.0	−1.8 ^c^ ± 0.1	−1.7 ^c^ ± 0.0	−1.8 ^c^ ± 0.0	−2.1 ^c^ ± 0.0	−2.3 ^c^ ± 0.1
5%WS20%ChE	0.4 ^a^ ± 0.0	−0.8 ^b^ ± 0.0	−0.8 ^b^ ± 0.2	−1.0 ^b^ ± 0.0	−1.2 ^b^ ± 0.0	−1.4 ^b^ ± 0.0
5%WS30%ChE	0.9 ^a^ ± 0.0	0.6 ^a^ ± 0.0	0.6 ^a^ ± 0.1	0.6 ^a^ ± 0.1	0.5 ^a^ ± 0.1	0.1 ^a^ ± 0.1
Gels with 7 °Brix extract						
L*						
5%WS (control)	57.5 ^a^ ± 1.0	65.7 ^a^ ± 0.0	66.3 ^a^ ± 0.1	67.1 ^a^ ± 0.2	67.7 ^a^ ± 0.5	69.5 ^a^ ± 0.5
5%WS10%ChE	35.6 ^b^ ± 0.1	41.6 ^b^ ± 0.1	41.7 ^b^ ± 0.0	41.9 ^b^ ± 0.1	42.5 ^b^ ± 0.3	42.9 ^b^ ± 0.1
5%WS20%ChE	31.3 ^c^ ± 0.0	35.9 ^c^ ± 0.1	36.5 ^c^ ± 0.1	36.3 ^c^ ± 0.1	36.9 ^c^ ± 0.2	36.9 ^c^ ± 0.5
5%WS30%ChE	28.8 ^d^ ± 0.1	31.2 ^d^ ± 0.1	31.5 ^d^ ± 0.0	32.4 ^d^ ± 0.5	32.3 ^d^ ± 0.1	31.8 ^d^ ± 0.6
a*						
5%WS (control)	−1.1 ^d^ ± 0.2	−2.4 ^d^ ± 0.0	−2.5 ^d^ ± 0.0	−2.4 ^d^ ± 0.0	−2.4 ^d^ ± 0.0	−2.5 ^d^ ± 0.0
5%WS10%ChE	16.9 ^a^ ± 0.3	19.2 ^b^ ± 0.1	19.2 ^b^ ± 0.0	19.0 ^b^ ± 0.0	18.7 ^b^ ± 0.3	19.5 ^b^ ± 0.1
5%WS20%ChE	15.6 ^b^ ± 0.1	20.2 ^a^ ± 0.1	20.2 ^a^ ± 0.0	20.0 ^a^ ± 0.2	20.0 ^a^ ± 0.3	20.2 ^a^ ± 0.6
5%WS30%ChE	12.9 ^c^ ± 0.0	16.3 ^c^ ± 0.1	16.3 ^c^ ± 0.1	17.1 ^c^ ± 0.6	17.1 ^c^ ± 0.1	16.4 ^c^ ± 0.0
b*						
5%WS (control)	−3.5 ^c^ ± 0.7	−6.2 ^d^ ± 0.0	−6.5 ^d^ ± 0.0	−6.3 ^d^ ± 0.1	−6.2 ^d^ ± 0.2	−6.6 ^d^ ± 0.0
5%WS10%ChE	0.8 ^b^ ± 0.1	−0.1 ^c^ ± 0.0	−0.4 ^c^ ± 0.0	−1.1 ^c^ ± 0.1	−1.2 ^c^ ± 0.1	−1.4 ^c^ ± 0.1
5%WS20%ChE	2.1 ^a^ ± 0.0	1.9 ^b^ ± 0.1	1.6 ^b^ ± 0.1	1.2 ^b^ ± 0.1	1.1 ^b^ ± 0.1	1.0 ^b^ ± 0.0
5%WS30%ChE	2.6 ^a^ ± 0.0	2.7 ^a^ ± 0.1	2.5 ^a^ ± 0.0	2.1 ^a^ ± 0.2	1.9 ^a^ ± 0.0	2.1 ^a^ ± 0.1

Parameters in columns for each parameter denoted with the same letters do not differ significantly at the confidence level α = 0.05.

## Data Availability

The data that support the findings of this study are available from the corresponding author upon reasonable request.

## Supplementary Materials

The following supporting information can be downloaded at: https://www.mdpi.com/article/10.3390/molecules30214213/s1.

## Abbreviations

The following abbreviations are used in this manuscript:

BUs	Brabender units
ChE	Chokeberry extract
WS	Wheat starch
